# Belonging home: capabilities, belonging and mental health recovery in low resourced settings

**DOI:** 10.1093/heapro/daaa006

**Published:** 2020-04-11

**Authors:** David Cappo, Brian Mutamba, Fiona Verity

**Affiliations:** 1 YouBelong Uganda, Plot 199, Natasha Road, P.O. Box 36510, Kampala, Uganda; 2 Clinical Department, Butabika National Mental Hospital, Butabika Road, P.O. Box 7017, Kampala, Uganda; 3 College of Human and Health Sciences, Singleton Park, Singleton, Swansea, UK SA28PP

**Keywords:** community-based mental health, deinstitutionalization, family, belonging, Uganda

## Abstract

There are significant barriers to the development of a ‘balanced model’ of mental health in low-income countries. These include gaps in the evidence base on effective responses to severe mental health issues and what works in the transition from hospital to home, and a low public investment in primary and community care. These limitations were the drivers for the formation of the non-government organization, YouBelong Uganda (YBU), which works to contribute to the implementation of a community-based model of mental health care in Uganda. This paper overviews an intervention protocol developed by YBU, which is a combined model of parallel engagement with the national mental hospital in Kampala, Uganda, movement of ‘ready for discharge’ patients back to their families and communities, and community development. The YBU programme is theoretically underpinned by a capabilities approach together with practical application of a concept of ‘belonging’. It is an experiment in implementation with hopes that it may be a positive step towards the development of an effective model in Uganda, which may be applicable in other countries. Finally, we discuss the value in joining ideas from social work, sociology, philosophy, public health and psychiatry into a community mental health ‘belonging framework’.

## INTRODUCTION

This is a commentary paper focused on a community mental health intervention in East Africa that supports people living with severe mental illnesses (SMIs) transition from hospital to home. The need for this intervention emerged from the convictions and lived experience of the Ugandan based authors, one a public health psychiatrist and the other a social worker, who in 2016, established YouBelong Uganda (YBU), a Ugandan-based non-government organization (NGO) as a ‘connector’, or ‘bridge’ between the national mental hospital in Uganda and the wider community. The YBU programme works simultaneously to support people return home and to reduce unnecessary hospital readmissions. It is not a stand-alone or siloed NGO providing mental health psycho-social services (albeit important and essential roles), but is a programme integrated into the functions of the national mental health hospital. In Uganda this work is urgent and complex owing to the scale of unmet needs, high re-admission rates and hospital overcrowding and scarce resources. A compounding factor is the limited availability of community-based responses (Gureje *et al.*, 2005; Cohen *et al.*, 2011; Caddick *et al.*, 2016). As Auspos and Cabaj [(Auspos and Cabaj, 2014), p. 5] write, implementing change in complex circumstances means attending to a ‘diverse and interrelated array of factors’.

## BACKGROUND

There are frameworks which set out a vision and blueprint for community mental health practice and policy (BasicNeeds, 2009; Cohen *et al.*, 2011; Thornicroft and Tansella, 2013; Caddick *et al.*, 2016; Thornicroft *et al.*, 2016). Established evidence emphasizes the importance of relationship or person-centred interventions, understanding the social determinants of mental health, implementing ‘good practice’ in hospital to home transitions and the provision of family support (Cohen *et al.*, 2011; Thornicroft *et al.*, 2011, 2016; Allen *et al.*, 2014; NICE Guidelines, 2016). The UK NICE Guidelines (NICE, 2016) for *Transition between inpatient mental health settings and community or care home settings* state that; hospital care needs to be ‘… for the shortest possible episodes’ (p. 6); attention must be on recovery and person-centred care (p. 12); people are to be active in their own discharge planning; engagement with those in the discharged person’s support networks is important (p. 12); social relationships and activities are crucial (p. 13) and health and social care services need to work together (p. 13). Furthermore, understanding the way people who use services interact with health and care systems is the basis for realistic planning, service design and effective delivery.

Thornicroft and colleagues developed an integrated mental health ‘balanced care model’ which emphasizes both hospital provision together with primary health care responses (Thornicroft and Tansella, 2013; Thornicroft *et al.*, 2011, 2016). In low-income countries faced with comparatively fewer resources and limited availability of tertiary and secondary mental health services, Thornicroft *et al.*, suggest such an approach would include;

Primary mental health care with specialist back up care, screening and assessment by primary health staff, talking treatments including counselling and advice, pharmacological treatment, liaison and training with mental health specialist staff, where available for training, consultation for complex cases, inpatient assessment and treatment for cases which cannot be managed in primary care, for example in general hospitals.

[(Thornicroft *et al.*, 2011), p. 110]

Notwithstanding the ambition for the development of a ‘balanced care model’ and the existing knowledge base, in low-income countries there are substantial challenges to putting this approach and knowledge into practice. The political, policy, social and economic context is not readily conducive to attaining these objectives. Resourcing these interventions is a challenge. Social stigma about mental health, and often associated with those who work in mental health, an irregular supply of essential medications, low levels of trained community mental health staff, and a reluctance, in part, of the health care sector to engage with mental health patients increases the complexity and dilemmas (Thornicroft *et al.*, 2016). These are magnified in the absence of strong public voices advocating for the promotion of mental health care, both the voices of mental health service users and their families and of an alternative discourse of prevention and primary health care.

Addressing the high level of social stigma and the cultural and spiritual context dominating community understanding of serious mental illness is paramount. This requires cultural change and systems change and the development of a community mental health service in Uganda. As many authors outline, SMI in Uganda is understood within culture as the action of ancestral spirits, demonic possession, or the result of curses, and witchcraft (Abbo *et al.*, 2008; Van Duijl *et al.*, 2014; Hecker *et al.*, 2015). Implicit in a balanced model of mental health is public education about different understandings of the causes of serious mental illness that are evidence based. Such an approach will have limited impact without a cultural framework, where cultural and linguistic understandings about mental illness are integral to the conceptualization of both the causes of severe mental illness (SMI) and evidence-based treatment and social support (Kohrt and Mendenhall, 2015). This is particularly true for low- and middle-income countries where the prevalence and pattern of persons with SMI attending traditional health practitioners reflects the community beliefs that people with SMI are afflicted by spirits or supernatural forces (Abbo *et al.*, 2008).

Set against appreciation of these realities and dilemmas, YBU set out to develop a model of community-based mental health recovery which places at its centre ‘belonging’ to family, local community and culture. In this commentary paper, we describe the resultant intervention and its underpinning theoretical framework that combines principles of ‘belonging’ and ‘capabilities’ with a family-centred support model that the authors contend is suited to a resource challenged setting such as Uganda. We begin with an overview of the context and background of mental health policy and practice in Uganda, before outlining the model of YBU and its implementation in more detail. We suggest that there are unique practical innovations in this YBU model; the close partnership between the national mental health hospital and the NGO, and the melding of values and practices that are informed by social work, philosophy, public health and psychiatry into a practical framework of ‘belonging/capabilities’. In this paper, we define an ‘intervention protocol’ as a framework for practice that defines values and principles, key knowledge bases and the practical tools for implementation and monitoring and review. We conclude with a research agenda that is underway to test the efficacy and refine the YBU community mental health programme tools.

### Mental health care in Uganda

Uganda is a low-income country with a land mass of 241 000 km^2^. In 2016, it had a population of 34.6 million people and a 3% projected annual population growth rate (Uganda Bureau of Statistics (UBOS) and ICF, 2017). In 2008, the World Health Organisation (WHO) attributed 5.3% of the ‘disease burden’ in Uganda to ‘neuropsychiatric disorders, conditions such as anxiety, schizophrenia and depression’ (WHO, 2008). The existing provision of mental health services in Uganda are guided by the *Draft Mental, Neurological and Substance (MNS) Use Disorder Policy (2010)* and *a new Mental Treatment Act (2018).* Less than 1% of the national health budget is allocated to mental health [(Molodynski *et al.*, 2017), p. 98]. Furthermore, in Uganda, as with many low-income countries in Africa, mental health services are predominately delivered by large mental hospitals, a legacy from the colonial era, which are stretched to capacity.

Apart from the National Mental Hospital at Butabika (a fully funded government facility), mental health is funded as an integrated component of primary health care at other levels of care (Kigozi *et al.*, 2010). The resources for community-based early intervention are limited leading to progression of symptoms of SMI and to chronic illness requiring intensive long-term treatment. Without community-based support, the families of a person with SMI and the police, can view the mental hospital as the default resource in responding to behaviour disorders, signs of ‘madness’ and for containment, shelter, food and family respite. YBU commissioned a mixed methods Baseline Study Report (Turiho *et al.*, 2018) to explore the profile of people admitted to the community mental hospital in Kampala (i.e. socio-demographic profile, health status), family and carer’s supports and ‘help seeking’ practices. A case file review for a period of a year (August 2016 to September 2017) revealed that 20.3% of people discharged had been readmitted. Some had been readmitted twice. It was also commonplace that people stayed longer in hospital than they needed to. Co-morbidity rates were high, with 40% of those admitted in the year also having an alcohol or substance use disorder (Turiho *et al.*, 2018).

Across Uganda, there are a few non-government psychosocial services and private mental health care for those who can afford it, but these are mostly in urban areas. There is no formal community-based mental health service provision, as is also the case in many countries in Africa (Thornicroft *et al.*, 2016), nor is there service integration or centrally planned or systemic approaches to the management of ‘inflow into and outflow from’ the mental health system. As Kigozi *et al.* (Kigozi *et al.*, 2010) suggest, the development of mental health care must acknowledge the importance of all these components and work to find their place in a working framework. In the following section of the paper we turn to an overview of the YBU model of family centred, community mental health care.

## THE YBU PROGRAMME—A FAMILY AND COMMUNITY-CENTRED MODEL

### Conceptualizing the model

The YBU approach to deinstitutionalization and building community-based supports is in keeping with evidence-based research, and with Ugandan Government Health Policy (Second National Health Policy, 2010). The model has distinct parts: a values base, an implementation plan which is focused on the use of pre- and post-hospital discharge tools and family and community empowerment work. In the development of the model, YBU has worked to harmonize a ‘balanced model of care’ with support for the training of the general primary health system in mental health, and initiatives such as task sharing and peer support, as advocated in the literature (Thornicroft *et al.*, 2011).

The work of YBU is influenced by the social determinants of mental health (WHO, 2014). The latter necessitates inserting the YBU model of care in a social, economic and cultural context, which means attention is given to levels of poverty and unemployment, food and housing, insecurity and education completion rates, as well as the impact of these experiences on families, care givers and communities. There are also strengths and assets within families and communities and recognition of these is a foundational aspect of the long tradition of empowerment in social work and community development (Lee, 2001). This incorporates and moves beyond a clinical framework to a social framework.

### Values and principles

As noted, YBU is informed by notions of empowerment and focuses on concepts of belonging to family, place, community and culture. These principles for praxis blend a philosophical position (Taylor, 1992) as well as a sociological position (May, 2013) in an understanding of what might best assist a recovery process for people with SMI. Recovery is viewed as a multi-dimensional concept. It is taken to mean a process and mindset (i.e. hopefulness, positive engagement in life and for long-term relationships, no matter how difficult) and often means living with and within limits of a mental health condition (Jacob, 2015).

Influenced by the writings of the philosopher Charles Taylor, YBU adopts aspects of communitarianism, seen in its commitment to a relational context for the development of personhood, and viewing the formation of self-identity, emotional well-being, values development, as occurring not in isolation from others, but in relation to and with other persons, and in particular within the structures of family, community and culture. From this position, comes the assertion that to promote well-being in general, and for those who have been marginalized or abandoned by family and community, a reconnection to family and local community and tribal culture, should be facilitated.

Belonging, as a key resource and process, is central to the intervention described in this paper. Belonging is deeply emotional, ontological and cultural (Vanier, 1975, 1998). In one sense to ‘belong’ is to be visible, recognized and engaged within social attachments or relationships (Yuval-Davis, 2006). In another sense, belonging is associated with human rights; they sit together (Hastings, 1997; Pettitt *et al*., 2016). People may belong or identify with a nation, community, family or ethnicity and culture, and with this ‘belonging’ come understandings and practices of rights and responsibilities. A distinction is drawn between what Garbutt (Garbutt, 2009) calls a relational and ongoing process of ‘connection and connecting’ and a fixed belonging to a social group or place (p. 84). Moreover, Garbutt writes belonging ‘… may also be constrained by a range of other considerations such as lack of transport, or poverty, or narrow constructions and representations of national identity’ (p. 88). Youkhana [(Youkhana, 2015), p. 11] tacks in a similar direction away from ‘… categories with inherent spatialities, territoriality, and boundary making to concepts based on movement and flow’. The complexity and multi-dimensions of ‘belonging’ are inherent in what Yuval-Davis calls the contours of the ‘politics of belonging’ (2006).

Experiences of ‘belonging’ are linked to mental health recovery (Doroud *et al.*, 2018). Belonging has been identified as a ‘mediating factor’ or ‘buffer’, between life experiences and mental health (Torgerson *et al.*, 2018), and strongly associated with positive mental health (Kitchen *et al.*, 2012; May, 2013). Conversely, feelings of not belonging are linked to poorer health outcomes (May, 2013). Doroud *et al.* (Doroud *et al.*, 2018) undertook a meta-synthesis of research that investigated place and social and mental health recovery, and observe ‘Place as a context for doing, becoming, and belonging emerged as central theme’ [(Doroud *et al.*, 2018), p. 112]. The importance of belonging and place will perhaps not be a surprise. The human quest and need to belong and belong home are as old as time. Homer’s epic tale *The Odyssey*, recounts the travails of Odysseus and his 20-year journey after a shipwreck to reach home, the island of Ithaca and family and community. Challenges and struggles along the way—the goddess Calypso’s distractions and the ‘thunder of the seas’—were eclipsed by the strength of the longing for home; ‘… to be back among his own people’ [(Homer, 1900), p. 14]. As Homer writes, Odysseus: ‘… is a man of such resource that even though he were in chains of iron he would find some means of getting home again’ [(Homer, 1900), p. 19].

### Practical implementation

Deinstitutionalization so people can return home is central to the work of YBU. Institutionalization in asylum like conditions is itself damaging to mental health and well-being. Overcrowding and high staff to patient ratios in the Ugandan national mental hospital, and extremely limited resources, with no resources for daily activity for in-patients, quickly leads to patients exhibiting the effects of institutionalization, such as lowered sense of personal agency, and motivation. Even when patients are ready for discharge, they remain in the ‘convalescent ward’ for extended periods of time (this timeframe can extend to many months, and in some cases years), due to the hospital’s limited resources to resettle people back to their families, or their families rejecting them and abandoning them, further ingraining the effects of institutionalization and triggering further decline in mental health. And for those patients who are re settled back with family, evidence would suggest that relapse and readmission to hospital is high (Butabika Hospital Records Department, 2017). Currently there are limited government resources to prepare the family for the patient’s return, nor support the family post-return.

YBU has positioned itself as a ‘bridge’ from the national mental hospital in Uganda, to the community. In practical terms, a memorandum of understanding has been established between the hospital and YBU, and a high level of trust and cooperation exists between both organizations. YBU has established itself with offices within easy access to the hospital. Professionals including social workers, psychiatric nurses and occupational therapists comprise the YBU community mental health team. A social worker and a psychiatrist lead the organization. YBU has its own transport provision for the return of persons to their homes and for access to families, a critical resource in a low-income country.

The first step in the YBU programme called YouBelongHOME, to assist in the deinstitutionalization process, is a family-centred mental health approach to engage with staff at the national mental hospital, in Kampala, Uganda, and to begin a process of movement of ‘ready for discharge’ patients back to their families and communities, and developing appropriate ways to support them in their communities. Throughout, an objective is to decrease potential for relapse and readmission to hospital (institutional) care. A set of tools have been developed to support this engagement work. The intervention is a tailored pre-discharge assessment tool, and post-discharge family-centred intervention tool. These tools have been influenced by a recent baseline needs study and are subject to testing as part of a research study underway at the time of writing (You Belong Uganda is a collaborating partner in a research study CHaRISMA, funded by the UK Department of Health and Social Care, the Department for International Development, the Medical Research Council and Wellcome.). They are the cornerstone of the YBU community model of care as described below.

#### Pre-discharge assessment tool

The pre-discharge assessment tool is designed as a checklist to be used by the YBU community mental health team, to talk with the person to be discharged, hospital staff, the family of the person to be discharged, and the local community, in which the person will reside. Through these guided conversations the assessment tool is designed to produce comprehensive data combining personal profile information, general health information, a wide-ranging mental health profile, and a profile that falls under the broad heading of the social determinants of mental health.

It is family centred in its approach, and mindful of the importance of ‘connecting and connections’ and emotional attachments for broad supportive relationships. It includes a family mapping exercise placed in the context of empowering the person returned home and their family and identifying needs to support the strengthening of person and family capabilities. It is also designed to highlight the role of the main carer in the family, and to identify the main carer’s needs, in strengthening the family as a unit. Frequently the main carer is a person in the family home who is prepared to care for the person and support them during recovery. The assessment tool has been designed *in situ* with a high level of cultural awareness.

#### Post-discharge intervention tool

The post-discharge intervention tool is placed in the context of an empowerment plan. The plan is prepared from the data collected in the pre-discharge assessment phase. The goals of the intervention plan are to develop and implement a family centred, community based, holistic and integrated plan for mental health recovery, to minimize the risk of relapse and readmission to institutional care, and to connect the person in recovery, and their family, to local community support and health care resources.

The three levels of the post-discharge plan of empowerment (in which families are encouraged to play an active part in its development) are shown below:

- *Support to returned family member*: First, to empower the family to support their returned from hospital family member by providing information and education on mental health care, stigma, including self-stigma, discrimination, problem solving skills, crisis management skills, conflict resolution, dealing with violence/aggression and problem behaviours, and effective communication skills. YBU is trialling an adapted form of family psycho education (FPE) known as Family Consultation (Thornicroft *et al*., 2011) as a co-produced evidence-based model to be used in the resource limited setting for YBU operations. Information about possible places to gain ongoing support are also discussed.
- *Building family capabilities*: Second, empowering the family is viewed as critical to the post-discharge intervention. The intention is to increase the family’s capabilities and opportunities, with particular focus on the health of all family members (including women and children), education opportunities for children, caring roles in the family (with particular attention to the role of the main carer), income generating opportunities (including possible access to micro grants), food, shelter and security.
- *Self-care capabilities*: Third, empowering and supporting the returned family member to move to a level of self-care, to maintain a medication regime, take charge of their recovery, and to participate in family and community life. Issues of stigma and the effects of institutionalization are also addressed.

Within the evidence-based framework of the FPE model of Family Consultation, and allowing for some adaptation due to limited personnel resources, transport difficulties, etc., following one assessment meeting with the person to be discharged from hospital, and two assessment meetings with the family pre-discharge, the empowerment plan of the Family Consultation process is planned and implemented over a 12-week period. This is divided into three ‘at home’ consultations with the family and person discharged, over a 6-week period, followed by 6-week period of structured phone support. At the end of the 12-week period, there will be a further ‘at home’ face to face visit and interview, and then referral to the newly trained health centre and community health workers. The post-discharge empowerment plan has been developed in a cultural aware and sensitive framework and is a working document open to regular review.

#### Primary health care system

As experience to date is showing, because of the level of cooperation and coordination between YBU, and the national mental hospital and the Ministry of Health in Uganda, and together with the use of the pre- and post-discharge assessment and intervention tools, the flow of ready to be discharged patients out of the hospital can be significantly increased, and they can be settled back with their families with a level of formal preparation and a plan of empowerment for their on-going recovery. An important component of the model is that the YBU community mental health team provides on-going support both within the family home, and by phone, as outlined above. In recognition of the need for education and training of the primary health system in mental health, YBU will also engage in primary health care education in the two regions in which YBU operates (Kampala and Wakiso districts). The hope is that this will contribute to improved access points for community-based mental health care for families and their family member returned from hospital care, as well as initial points of access to evidence-based mental health for those with symptoms of mental illness rather than seeking care at the tertiary national mental hospital as the first (and only) option.

This undertaking is not without its complexity. Incentivizing, and education in mental health is fundamental to any plan to broaden the role of the primary health system with regards to mental health service provision. As noted earlier, this needs to be placed in a cultural context where culture and the language of culture is central in these processes. This is essential if education and training in mental health is to have lasting impact.

#### Educating the police in mental health

A key stakeholder in the Uganda mental health system is the national police force. In the YBU model they are considered an important adjunct to the system of care, and a potential partner in shifting the model of mental health care towards community-based services. Apart from the families themselves, they are a group who can identify behaviours that, in their opinion, warrant detention and movement, often with the use of force, and against the person’s will, to the national mental hospital. The police can see the national mental hospital as their only option in responding to behaviours they would consider unacceptable in the community, usually due to violence and unpredictability. It is their default position. The police can become major players in shifting the culture and behaviour of the population, away from viewing hospital care as the only possible option for care for mental illness. Providing educative and training programmes in mental health for the police, within a cultural context, and skill development, particularly in crisis management, in the two regions of YBU operations, is a key strategy of YBU.

#### Supply of medication

A common cause of relapse and readmission to hospital care, is the frequent interruption in the supply of psychotropic and other mediations (Thornicroft *et al.*, 2016). The unavailability of essential mediations, especially at lower level health facilities, quickly destabilizes many people in their recovery process within community. The reason for such interruption of supply can be caused by government budgetary constraints, inefficient bureaucracy or corruption. Maintaining people in recovery in the community, and minimizing relapse and return to hospital care, necessitates uninterrupted supply of medication for people with SMI. Dialogue with the Ministry of Health and other stakeholders, about this barrier to people’s recovery process is part of our work at YBU. In this short term, YBU is exploring the possibility of securing its own supply of essential medications for emergency use when government supplies are interrupted.

#### Further innovations

Further initiatives under development include the establishment of emergency response teams, that would support coordination of police, peer support workers, YBU workers and primary health workers, to respond to specific emergencies related to designated ‘high risk of relapse’ persons. As well YBU is exploring income generating work to increase the family’s social and economic participation in community. The latter reflects the relation between poverty and mental health (WHO, 2013). These micro-enterprise initiatives have an evidence base. Purchase of a goat, chickens, seeds for planting or textiles for craft work can have significant impact in generating long-term incomes. However, as Stites and Bushby [(Stites and Bushby, 2017), pp. 23–24] note, this evidence base is ‘mixed’ with varying assessment of who benefits from such initiatives. In some situations where micro-financing has been employed, benefit has been skewed to the micro-finance lenders. These initiatives need thoughtful planning and analysis of their known and unintended consequences, and analysis of sustainability and impact (Shaw, 2004).

The key values and components of YBU and the praxis for work are shown in Box 1.

## CONCLUSION

YBU is developing a community focused model of integrated mental health care. It is doing this within an environment of very limited resources in a low-income country, and a mental health system that is currently locked into hospital-based care (often in asylum like conditions) with negligible community-based mental health services. To address these complex challenges, YBU is adopting a multi-disciplinary approach both at the level of conceptual development and implementation services, as described above.

The NICE guidelines (NICE, 2016) are particularly helpful in isolating key components of a balanced system of integrated care. Together with the focus on the family as a key agent in a newly developed mental health approach in Uganda, and the use of the relational model of belonging as well as an orientation to cultural sensitive interventions, YBU is attempting to maximize personal and family resources, besides its work to support current community health services to take on a role in mental health services. YBU works to integrate short-term use of hospital care within a community focused system of care. By adopting the role of a ‘bridge’ between the national mental hospital in Uganda, and the family/community, YBU is working to be an influence in the movement of ‘patients’, i.e. the inflow of people into the mental hospital and the outflow from the hospital. It has a goal of reducing readmission to hospital care (now under research), and with the support of the Government Ministry of Health, to assist in building mental health resources in community, and in the existing health care system in Uganda.

The purpose of this endeavour is to contribute to systems change that will hopefully see tentative steps towards decentralized community mental health services, and the movement of the national mental hospital from an institutional model to that of an acute inpatient facility for specialized, short-term care. Limitations of this work to date are the lack of longitudinal data that the model is effective in achieving its ambitions. That said, there is on-going research to test the model and provide evidence of feasibility, acceptability and utility. YBU is a partner in a research project exploring the efficacy of the model, which will report in 2019. Findings from this study will contribute to clearer understandings about whether the practice developments extolled in this paper as innovative can deliver real and sustained benefits over time.

**Box 1:** Values/principles for praxis in YB work in Uganda

1. Personal identity is formed through personal and social relationships, particularly in and with family, local community and tribal culture.

2. The need to belong to family, community and culture, is a basic human need. Belonging is a resource and process essential for personal well-being, self-respect, recognition as a unique individual and as a member of the group, and for building personal dignity. Belonging is linked to mental well-being.

3. Recovery from SMI is a process with the active participation of, if not leadership by, the person in recovery. This process is built on hope and a conviction that in a supportive environment of family, community and culture, and with life adaptions, a meaningful and productive life in family and community is achievable.

4. Community-based mental health services should be the centre piece of mental health care, providing the person in community with ease of access to care ‘in community’. Acute in-patient care should only be used to stabilize the person experiencing behaviours associated with SMI, and this should be short term, leading to referral to community-based services for long-term support.

5. Provision of community-based services and supports, including innovative practices, should only be implemented if they are of high quality, there is potential for scale for significant impact in the community, and there is high possibility of sustainability.

6. The family unit, the local community from which a person recovering from SMI comes, and tribal culture (in Uganda and other African countries) are essential resources in the recovery process. Each play an important role in responding to the belonging needs of the person in recovery.

7. Building opportunities and skills for families, particularly of the main carer in the family empowers families and strengthens their capacity to respond to the range of issues associated with having a family member recovering from SMI.

8. Cultural context, awareness and sensitivity should be the core driver in education of families and communities in mental health. The strong cultural depth of understanding and interpreting mental illness in Uganda, particularly regarding the role of traditional and faith healers, and beliefs in ancestral spirits, curses, demons and possessions and the language used to convey these understandings, needs to be addressed within a cultural framework.

9. The decentralized health system in Uganda is a key resource. Primary health care workers, at multiple levels in the system need to be trained in mental health, particularly in WHO MH GAP. Community access points should be developed in the health system for families and individuals to receive community-based mental health services.
